# Supplementary material for: Resolving the full spectrum of human genome variation using Linked-Reads
Source: Genome Res. 2019 Apr;29(4):635–45. doi: 10.1101/gr.234443.118 (PMC6442396; doi:10.1101/gr.234443.118)
Supplement: Supplemental Material [file supp_gr.234443.118_Supplemental_longranger-2.2.zip › longranger-2.2.tar.bz2!/lr2.2-public/lib/stan/cmdstan/stan/doc/api/html/namespacemembers_func_s.html]

Stan: Namespace Members


|  |  |
| --- | --- |
|  | Stan  2.5.0  probability, sampling & optimization |


- Main Page
- Related Pages
- Namespaces
- Classes
- Files

- Namespace List
- Namespace Members

- All
- Functions
- Variables
- Typedefs
- Enumerations
- Enumerator

- a
- b
- c
- d
- e
- f
- g
- h
- i
- j
- l
- m
- n
- o
- p
- q
- r
- s
- t
- u
- v
- w


All Classes Namespaces Files Functions Variables Typedefs Enumerations Enumerator Friends Macros Pages

### - s -

- sample()
  : stan::common
- scaled\_add()
  : stan::math
- scaled\_inv\_chi\_square\_ccdf\_log()
  : stan::prob
- scaled\_inv\_chi\_square\_cdf()
  : stan::prob
- scaled\_inv\_chi\_square\_cdf\_log()
  : stan::prob
- scaled\_inv\_chi\_square\_log()
  : stan::prob
- scaled\_inv\_chi\_square\_rng()
  : stan::prob
- sd()
  : stan::agrad
  , stan::math
- segment()
  : stan::math
- set\_zero\_all\_adjoints()
  : stan::agrad
- sign()
  : stan::math
- simplex\_constrain()
  : stan::prob
- simplex\_free()
  : stan::prob
- sin()
  : stan::agrad
- singular\_values()
  : stan::math
- sinh()
  : stan::agrad
- size()
  : stan::math
- size\_of()
  : stan
- skew\_normal\_ccdf\_log()
  : stan::prob
- skew\_normal\_cdf()
  : stan::prob
- skew\_normal\_cdf\_log()
  : stan::prob
- skew\_normal\_log()
  : stan::prob
- skew\_normal\_rng()
  : stan::prob
- softmax()
  : stan::agrad
  , stan::math
- sort\_asc()
  : stan::agrad
  , stan::math
- sort\_desc()
  : stan::agrad
  , stan::math
- sort\_indices\_asc()
  : stan::math
- sort\_indices\_desc()
  : stan::math
- sqrt()
  : stan::agrad
- sqrt2()
  : stan::math
- square()
  : stan::agrad
  , stan::math
- squared\_distance()
  : stan::agrad
  , stan::math
- stan\_print()
  : stan::agrad
  , stan::math
- start\_nested()
  : stan::agrad
- step()
  : stan::agrad
  , stan::math
- student\_t\_ccdf\_log()
  : stan::prob
- student\_t\_cdf()
  : stan::prob
- student\_t\_cdf\_log()
  : stan::prob
- student\_t\_log()
  : stan::prob
- student\_t\_rng()
  : stan::prob
- sub()
  : stan::math
- sub\_col()
  : stan::math
- sub\_row()
  : stan::math
- subtract()
  : stan::math
- sum()
  : stan::agrad
  , stan::math
- suppress\_warning()
  : stan::gm


---

[ Stan Home Page ]

*© 2011–2014,
Stan Development Team.*
